# Production of Low Cost Carbon-Fiber through Energy Optimization of Stabilization Process

**DOI:** 10.3390/ma11030385

**Published:** 2018-03-05

**Authors:** Gelayol Golkarnarenji, Minoo Naebe, Khashayar Badii, Abbas S. Milani, Reza N. Jazar, Hamid Khayyam

**Affiliations:** 1 School of Engineering, RMIT University, Melbourne, VIC 3001, Australia; gelayol.golkarnarenji@rmit.edu.au (G.G.); reza.jazar@rmit.edu.au (R.N.J.); 2 Institute for Frontier Materials, Carbon Nexus, Deakin University, Waurn Ponds, VIC 3216, Australia; minoo.naebe@deakin.edu.au; 3 School of Engineering, Deakin University, Waurn Ponds, VIC 3216, Australia; khashayar.badii@deakin.edu.au; 4 Materials and Manufacturing Research Institute, University of British Columbia, Kelowna, BC V1V 1V7, Canada; abbas.milani@ubc.ca

**Keywords:** limited data, complex manufacturing systems, support vector machines, Artificial Neural Network, intelligent optimization techniques, system identification

## Abstract

To produce high quality and low cost carbon fiber-based composites, the optimization of the production process of carbon fiber and its properties is one of the main keys. The stabilization process is the most important step in carbon fiber production that consumes a large amount of energy and its optimization can reduce the cost to a large extent. In this study, two intelligent optimization techniques, namely Support Vector Regression (SVR) and Artificial Neural Network (ANN), were studied and compared, with a limited dataset obtained to predict physical property (density) of oxidative stabilized PAN fiber (OPF) in the second zone of a stabilization oven within a carbon fiber production line. The results were then used to optimize the energy consumption in the process. The case study can be beneficial to chemical industries involving carbon fiber manufacturing, for assessing and optimizing different stabilization process conditions at large.

## 1. Introduction

Composite materials are becoming increasingly important in a wide range of industries. An advanced composite material is made of a fibrous material system, such as carbon fiber yarns embedded in a resin matrix; therefore, the production process of carbon fiber and its properties are the main keys to high quality and low cost composites. Carbon fiber as a part of advanced composite materials is widely used in industries. Among the precursors used for the production of carbon fibers, polyacrylonitrile (PAN)-based precursors are the most common precursors. Stabilization, carbonization, and graphitization are the three consecutive steps in carbon fiber production. Temperature, Time, and Tension (TTT) are the main controlling parameters in the thermal stabilization process. Stabilization step is the most complex, costly and energy consuming step. Hence, production quality and energy optimization of this step are deemed important to produce high quality and low cost composites. The stabilization oven is normally divided into four zones, each set at different temperature regions, which can be studied as individual systems. This is due to the fact that each zone has its own effect on the properties of resultant carbon fiber. In industrial settings, conducting experiments for all system parameters is often impractical due to the technical and time limitations, and this would make intelligent mathematical models a critical step for reliable process optimization purposes with limited datasets. Intelligent modeling techniques, such as stochastic [1,2] and heuristic [3], are powerful tools to analyze complex nonlinear systems, such as the stabilization process. Stochastic techniques need to have a number of experimental data compare to heuristic techniques. Such heuristic techniques as artificial neural network (ANN), adaptive neuro-fuzzy inference system (ANFIS), fuzzy logic, stochastic models, and support vector regression (SVR) with black box characteristics, have shown great generalization ability through different engineering case studies [4,5,6,7]. One of the frequently used modeling systems in the current literature is the Support Vector Machine (SVM) that is developed by Vapnik [8,9]. Another long-known method is the ANN that provides a viable modeling framework when complex nonlinear relationships exist between the inputs and outputs of a given system [1,2,3,10,11,12,13,14,15,16]; however, they have not been applied methodically to the second zone of thermal oxidative stabilization process. In terms of production quality (chemical, physical, and mechanical properties of oxidized PAN fiber (OPF) and carbon fiber), a few investigations have been reported in the literature. Dynamic models were used to study and analyze the carbon fiber thermal stabilization process [3]. Taylor polynomial method, Gauss-Newton algorithm (GNA), Levenberg-Marquardt algorithm (LMA) neural network, and genetic algorithms were used and compared. Based on the results, a given PAN fiber heat of reaction can be optimized by appropriate values of temperature and heating ramp. Khayyam et al. [2] also developed a stochastic optimization model for energy management in the carbonization process of carbon fiber production line. Their study indicated that it is possible to use stochastic optimization models to predict the production quality and minimize the energy consumption of its industrial process. Badii et al. [13] developed a model to predict density based on PAN precursor and OPF functional groups. Golkarnarenji et al. [17] developed an intelligent predictive model for energy consumption in thermal stabilization process, considering production quality and controlling stochastic defects. That study, however, was carried out for the first zone of stabilization process. Regarding predictive modelling for energy consumption in industrial processes, there are also some studies that are available in literature [18,19,20]. None of the aforementioned studies on energy optimization is based on density in the second zone of stabilization process. Hence, the aim of this article is to test the ANN and SVR techniques against forecasting physical properties of the OPF, with a limited dataset available from the second zone of stabilization oven. The result of this prediction was used for optimization of oxidative stabilization process to reduce the energy consumption and ultimately cost. Different optimization techniques can be used to optimize energy consumption and find the optimal process condition for carbon fiber manufacturing [21,22,23,24,25]. Genetic Algorithm (GA) was selected in this study.

The main objectives and contributions of the present manuscript are:

- Develop a model for physical property of the OPF (here density) in the second zone of stabilization process, which can then be considered in the optimization of energy consumption in the process.
- Develop a surrogate model for energy consumption and management.
- Optimize the energy consumption in the stabilization process, given the range of process constraints, such as fiber density.

The rest of this paper is organized as follows. In Section 2, the experimental procedure is presented. Section 3 discusses the modelling framework for physical property of carbon fiber and energy management in the stabilization process. Section 4 presents the results of predictive models, energy consumption in stabilization process and its optimization. In Section 5, concluding remarks are presented.

## 2. Materials and Methods 

**Figure 1:**
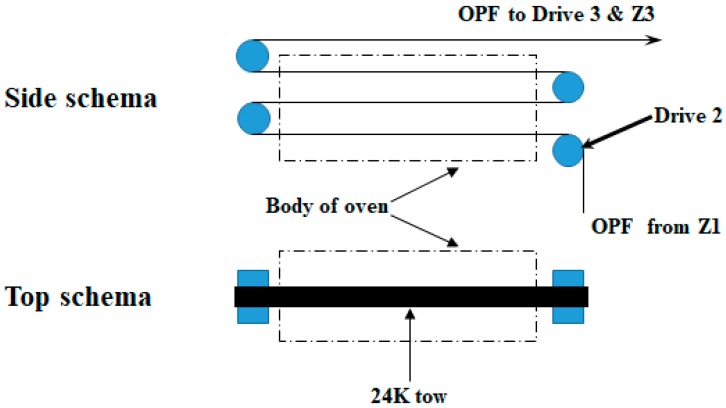
Schematic diagram of zone 2 of the stabilization oven studied.

The PAN precursor used in the study was a commercial fiber provided by the Blue Star (China) enterprise with a density of 1.1993 g·cm^−3^ and linear density of 1.58 dtex. The ANN and SVR modelling were carried out for select combination of the following operational conditions during stabilization of PAN: temperature (five temperatures between 233 and 241 °C, including 233, 235, 238, 239, and 241 °C, based on manufacturing experiences), space velocity (20, 30 and 35 m/h), and stretching-ratio (1.0%, 2.0%, 3.0%, and 4.0%) in the second zone of single tow oxidation pilot oven, designed by Despatch industries. Figure 1 shows the schematic diagram of the second zone of stabilization oven. 13 tests were used for training purpose and three additional tests were performed to validate the developed models. The tests were randomly designed to prevent any prejudgment. The density (g·m^−3^) of the fiber samples was measured with the density gradient technique [26]. The stretching ratio was controlled by the difference percentage between speed of Drive 1 and Drive 2. 

## 3. Modeling Framework

### 3.1. Support Vector Regression (SVR)

The Support Vector Regression (SVR) is a part of Support Vector Mechine (SVM) that is designed to find unique global solutions to curve fitting problems with highly nonlinear data. The SVM models are commonly used to solve both classification and regression problems. In this method, the data is mapped into a higher dimensional feature space and is fitted to a linear function with minimum complexity using kernel functions [27,28]. Three common kernel functions in SVR are the sigmoid kernel function, the polynomial kernel function, and the Gaussian RBF kernel function. In this article, the Gaussian RBF was selected [29] and the value of C which is the trade-off between error minimization and margin maximization, the value of ε to build the regression function by managing the number of support vectors, and the parameter γ in RBF is to be optimized using GA. Given train dataset {(*x*1,*y*1), (*x*2,*y*2), ..., (*x*n,*y*n)}, SVR defines a function for the relationship between *x* (input) and *y* (output) as:

(1) $$f\left(x\right)=\sum_{i=1}^{n}w\varphi \left(x\right)+b,$$

where *x* is mapped to a new space by *φ*(*x*) when the input-output relation is nonlinear. In the new space, the relationship of *φ*(*x*) and *y* is linear. A linear hyperplane is then determined by the variables *w* and *b* that can be fit to the training dataset. The goal of SVR solution is to lower the expected risk. This has been presented in Equation (2).

(2) $$R_{emp}=\frac{1}{n}\sum_{i=1}^{n}L_{\varepsilon }\left(y_{i},f\left(x_{i}\right)\right),$$

where *L*_ε_ is ε insensitive loss function per Equation (3).

(3) $$L_{\varepsilon }\left(y,f\left(x\right)\right)=\{\begin{array}{l} 0,\;if\;\left|y-f\left(x\right)\right|\leq \varepsilon \\ \left|y-f\left(x\right)\right|-\varepsilon ,\;Otherwise \end{array},$$

SVR uses linear regression to minimize the expected risk using ε- insensitive loss function and minimizes $\left|\left|w^{2}\right|\right|$ to reduce the complexity of the model. This has been shown in Equation (4).

(4) $$\underset{w,b,\xi ,\xi^{*}}{\min}\frac{1}{2}\left|\left|w^{2}\right|\right|+C\sum_{i=1}^{n}(\xi_{i}+\xi_{i}^{*}),$$

Subject to:

$$\{\begin{array}{l} w\phi \left(x_{i}\right)+b-y_{i}\leq \varepsilon +\xi_{i},\\ y_{i}-w\phi \left(x_{i}\right)-b\leq \varepsilon +\xi_{i}^{*},\\ \xi_{i}^{*},\xi_{i}\geq 0,\;i=1,2,\ldots ,n. \end{array},$$

where $\xi_{i}$, $\xi_{i}^{*}$ (*i* = 1, …, *n*) are the non-negative slack variables that show the difference between the *f*(*x*) and real value of training data.

This optimization problem can be converted to a dual problem, as in Equation (5).

(5) $$f\left(x\right)=\sum_{i=1}^{n}\left(a_{i}^{*}-a_{i}\right)K(x_{i},x)+b\;where:\;0\leq a_{i}^{*}\leq C\;\&\;0\leq a_{i}\leq C,$$

$a_{i}$ and $a_{i}^{*}$ are the Lagrange multipliers obtained from the dual problem. $K\left(x_{i},x_{j}\right)$ is determined by the inner product of *φ*($x_{i}$) and *φ*($x_{j}$), which is the kernel function. In this study, RBF was selected as the kernel function, which is represented by Equation (6).

(6) $$k\left(x,z\right)=\exp\left(\frac{{\left||x-z|\right|}^{2}}{2\gamma^{2}}\right),$$

### 3.2. Artificial Neural Network (ANN)

ANN is a highly developed machine learning methodology that has been used in several areas of prediction, control, and process identification [30]. The ANN consists of neurons connected to each other. Neurons are positioned in layers and each layer’s neurons function in parallel. An input layer, hidden layers, and an output layer compose the ANN architecture [31]. Key parameters in ANN are the interconnection of different neuron layers, the updating the weights of the interconnections, training algorithm and activation function. Both the learning error and the prediction error should be minimized by a good artificial network. Some form of gradient descent, using backpropagation is employed by most training algorithms to calculate the function gradients. The common training algorithms for ANN include the Scaled Conjugate Gradient (SCG), Bayesian Regularization (BR), and LM (Levenberg-Marquardt) [32,33]. SCG algorithm needs less memory; however, it has good generalization for noisy datasets. BR typically takes more computational time. LM algorithm [32,34] has good convergence properties. It takes more memory but less computational time; it is the fastest back-propagation algorithm for training and prediction purposes. This algorithm was also used in the current study.

### 3.3. Performance Validation

The standard error of prediction (SEP) was primarily used to calculate the performance of each predictive model based on the following equation:

(7) $$SEP=\sqrt{\frac{\sum {\left(y_{exp}-y_{pred}\right)}^{2}-N{\left(\sum \left(y_{exp}-y_{pred}\right)\right)}^{2}}{N-1}},$$

where *N* is total sample size and $y_{exp}\;\mathrm{and}\;y_{pred}$ represent the experimental and predicted dependent variables, respectively.

In addition to SEP, the coefficient of variation (CV) was also used for the performance validation purposes, as follows:

(8) $$CV_{\left(n\right)}=\frac{1}{n}\sum_{i=1}^{n}MSE_{i},$$

where $MSE_{i}=$ ${\left(y_{exp_{i}}-y_{pred_{i}}\right)}^{2}$.

### 3.4. Energy Sources and Model Structure

Electrical heater for air, recycling fan, exhaust fan, drives (drives 1 and 2 for the second oven), and PAN fiber tow are sources that consume energy (Figure 1). Energy release occurs during the thermal stabilization process. This release is fast and should be taken in to consideration as it increases the possibility of ignition or even combustion. Nonetheless, this release of energy is neutralized and controlled by cold “makeup” air from outside to uniform the temperature inside the oven.

## 4. Results

Two models were developed for prediction of the OPF density (g·m^−3^), based on the selected main controlling process parameters: temperature (°C), space velocity (m/h), and stretching ratio (%). In order to predict density based on these parameters, 16 experimental data were randomly distributed into two subsets (70% for training and 30% for testing): 13 for model development and 3 for comparison with experimental data (validation). In terms of data pre-processing, the predictors were normalized to the popular bound between 0 and 1 to improve the model identification performance [35]. To develop a fitting model, the ANN, and SVR, Matlab (Mathworks, Natick, MA, USA) were employed. The leave-one-out cross–validation technique was used to ensure the lack of overfitting. Based on the calculation of CV and SEP, the fitted models were evaluated. For ANN, Fitnet was used as the function fitting feed forward neural network. In order to train the network, different methods were tested and finally the Levenberg–Marquardt function (trainlm) was selected. The configuration of developed ANN model has been shown in Table 1.

**Figure 2:**
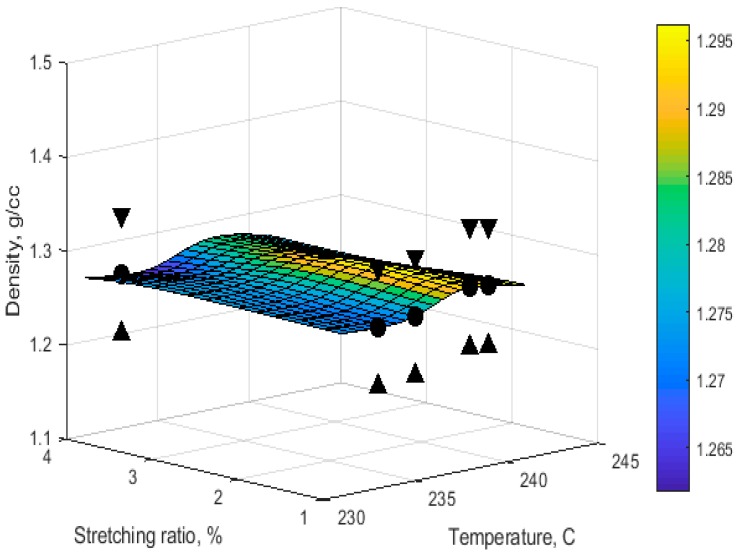
Temperature-stretching ratio relationship with density at space velocity of 20 m/h using Artificial Neural Network (ANN); the triangle marks show the prediction error.

After a number of trials, a neural network with three inputs (temperature, space velocity, and stretching ratio), one intermediate hidden layer with nine neurons and one output (density) was configured. A tangent sigmoid (tansig) transfer function was used within the hidden layer, and a linear (purelin) transfer function was used in the output layer. The CV and SEP obtained were 0.0000149, and 0.0037, respectively. Figure 2 shows the example of the predicted relationship between the density, stretching ratio, and temperature parameters with the space velocity of 20 m/h in the ANN model.

Next, a SVR model was developed as an alternative to the ANN approach for the prediction of density. Since the general ability of SVR is significantly influenced by the modeling parameters, *C*, γ, and ε, the GA was used to optimize these values globally. The lowest fitness value was selected based on MSE. Table 2 shows the optimized values for *C*, γ, and ε. Similar to the ANN case, the dataset was divided into training and testing subsets, 13 samples for training and 3 samples for testing. For training the SVR, the leave-one-out cross–validation technique was used given the limited dataset.

**Figure 3:**
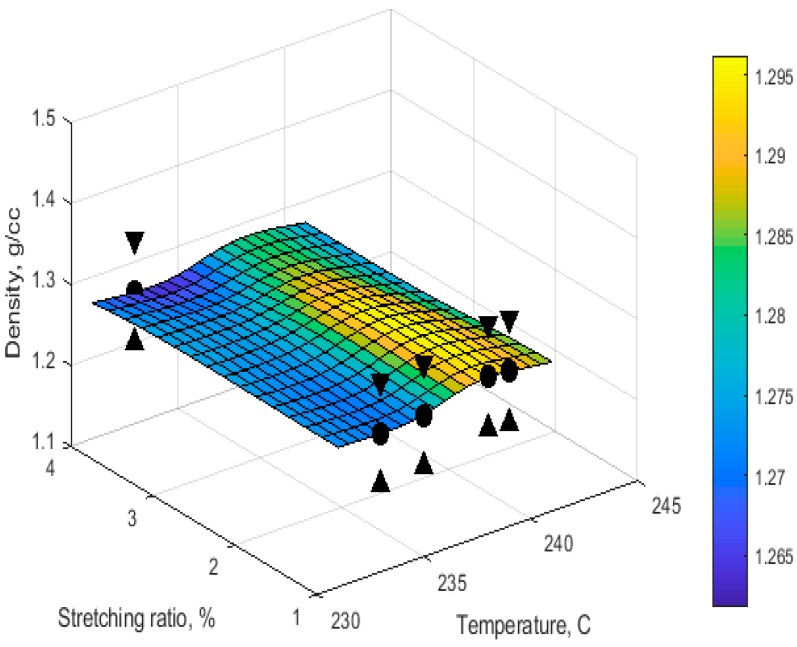
Temperature-stretching ratio relationship with density at space velocity of 20 m/h using Support Vector Regression (SVR); the triangle marks show the prediction error.

The Gaussian RBF [36] was selected as the kernel function for SVR prediction. In addition, to test the accuracy of the model, CV, SEP was again chosen as performance criteria. The CV and SEP were 0.0000117, 0.0019, respectively. Figure 3 shows the predictability of the SVR model in sample space velocity.

### 4.1. Predictive Model Validation

Table 3 presents the comparison of the validation results of SVR and ANNs. The results illustrate that the SVR model has had a superior performance over ANN-LMA in the current case study with the limited dataset.

### 4.2. Optimization of Energy Consumption

As mentioned in Section 2, electrical heater for air, recycling fan, exhaust fan, and drives (Drives 1 and 2 for the second oven) were the sources that consumed energy. The energy release of the PAN fiber tow was not considered due to higher amount of hot air flow, compared to the size of fiber tow. The amount of released energy from fiber would also have no significant effect on the total energy balances in thermal stabilization system and it was ignored in the calculation procedure of optimization. The temperature of the oven was assumed to be uniform as the system is close to an isothermal state, particularly in the central part of the oven. In the conducted set of experiments, the maximum power of three phased electrical heater was 32,000 W, with a maximum current of 44.5 A, and the voltage of 415 V at 50 Hz. The currents for the exhaust fan, recycling fan, Drive 1, and Drive 2 were 1.5, 5, 0.4, 0.4 A, respectively, based on PLC (programmable logic controller) data logger under steady state condition. The difference of current was negligible between the two drives. The air flow was constant and fully developed turbulent flow.

**Figure 4:**
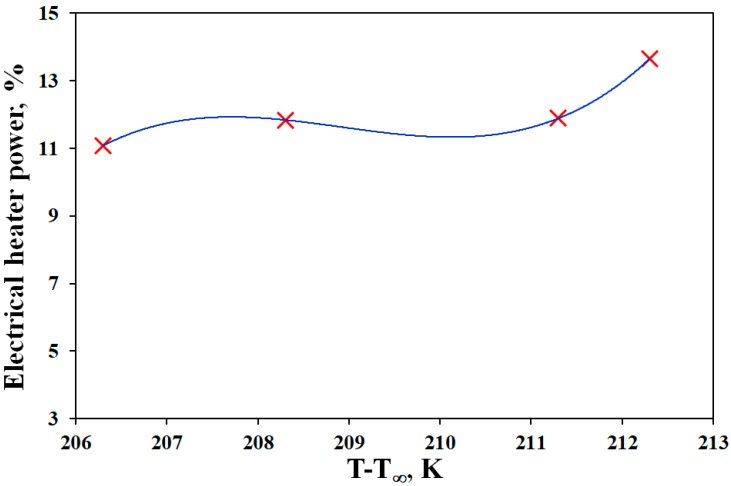
Fitted data for the power of electrical heater× as a function of difference between reactor and ambient temperatures; (×) experimental data and (▬▬) fitted curve.

To estimate the energy consumption of the recycling fan, exhaust fan, drives (Drives 1 and 2) for the second oven, Equation (9) was used [17].

(9) $$P=\sqrt{3}\times V\times I\times \left|\cos\varphi \right|,$$

where *P*, *V*, *I*, and *φ* are power (W), voltage (415 volts), current (A), and phase difference (120°), respectively. In order to estimate the energy consumption of electrical heater for air, Badii et al. [37] explained the structural details of this electrical heater. The power was determined based on the difference between the oven and the ambient temperature. It was assumed that the power of heater was only a function of this difference, as heater and make-up air are the only sources of energy. To predict the power for different levels of temperature, the PLC data was used under steady state conditions. As it has been shown in Figure 4, the data did not follow a linear trend and to find the relationship between the temperature and consumption of energy in the heater, minimum of four data points were used. The PLC data were recorded in steady state condition and the data of power was fitted to a polynomial equation (Equation (10)). The R^2^ value was 0.9999 for this equation.

(10) $$\%\Delta P_{EH}=-772723.770399017+11096.931232499\Delta T-53.117691666662\Delta T^{2}+0.08475\Delta T^{3},$$

where

(11) $$\Delta T=T_{r}-T_{\infty },$$

$T_{r}$ and $T_{\infty }$ are the oven and ambient temperatures, respectively. The average temperature of ambient was ~26.7 °C during the test period. The results of Equation (10) were multiplied by 32,000 (total power of heater, *W*) and divided by 100 (to change percentage to proportion) to predict the power of heater. To change the power to energy, the results were also multiplied by 3600 (to change time from hour to second), length of tow (*l*), and divided by the space velocity of fiber.

The best model obtained for energy consumption was:

(12) $$E\left(T,S,\sigma \right)=\frac{3600\times L\times \left(P_{Oven}\right)}{S}+\frac{\sqrt{3}\times 3600\times V_{F_{1}}\times I_{F_{1}}\times L\times \left|\cos\varphi \right|}{S}+\;\frac{\sqrt{3}\times 3600\times V_{F_{2}}\times I_{F_{2}}\times L\times \left|\cos\varphi \right|}{S}+\frac{\sqrt{3}\times 3600\times V_{D}\times I_{D}\times L\times \left|\cos\varphi \right|}{S},$$

where *T* is temperature (°C), *S* is the space velocity (m/h), σ is stretching ratio (%), *E* is the total energy consumption in joules for 6 m of fibers, *L* is length of tow (*L* = 3 × 2 m), $P_{Oven}$ is power of electrical heater, and $V_{F_{1}}$, $I_{F_{1}}$ $V_{F_{2}}$, $I_{F_{2}}$, and $V_{D},I_{D,}$ are the voltage and the current of recycle fan, exhaust fan, and drives, respectively. 

The energy consumption (Equation (12)) was then minimized using GA with a population of 20 individuals. In order to optimize the energy of the oven, Equation (12) should be optimized as:

Minimize E(T,S,σ), subject to:

$$\left\{ \begin{array}{l} S_{min}\leq S\leq S_{max}\\ T_{min}{}^{\circ}C\leq T\leq T_{min}{}^{\circ}C\\ \sigma_{min}\leq \sigma \leq \sigma_{max}\\ \rho \approx Svr\;model \end{array}\right.$$

*S* is the is the space velocity, $S_{min}$ is 20 m/h and $S_{max}$ is 35 m/h. $T_{min}$ is 233 °C and $T_{max}$ is 241 °C. σ is stretching ratio. $\sigma_{min}$ is 1% and $\sigma_{max}$ is 4%. The desired range of density was considered (1.27 g·cm^−3^ ≤ ρ ≤ 1.29 g·cm^−3^) based on the manufacturers demand. Table 4 presents the amount of energy consumption for each experimental condition.

**Figure 5:**
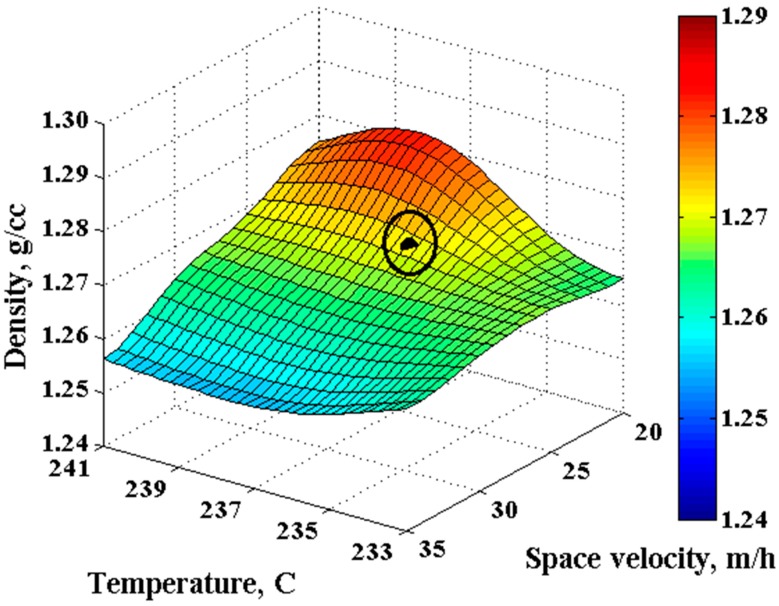
Position of the optimized energy criteria based on density constraint of 1.27 g/cm^3^ ≤ ρ ≤ 1.29 g/cm^3^.

Table 5 presents the results of the optimization process based on the given constraints, using GA method as the optimizer. The obtained optimum operational conditions were tested using additional experiments and the prediction error (Table 5 and Figure 5) was less than 5%, confirming an excellent agreement between the optimization model and experiments. Figure 5 shows the results of energy optimization based on density as a constraint. The results of the model for density of OPF have been presented in this figure as the main parameter for controlling the quality of OPF. The black point is the result of optimization. The energy consumption was 5.513 MJ (seventh column in Table 5) to achieve the density constraint (the first column in Table 5). These results show up to −48.6% energy saving by the developed model (the last column in Table 5). Elapsed time was 61.99 seconds for this system. The error for predicted density (fifth column of Table 5) is ~1.7% (ninth column of Table 5). Actual density (experimental) results are shown in the sixth column of Table 5.

## 5. Conclusions

The study of stabilization process, as the most energy consuming part of the carbon fiber production line, is the key to optimize the energy and cost during production of carbon fiber-based composites. In order to fulfill this purpose, the SVR and ANN models were tested in a case study to predict the density of OPFs based on the main controlling parameters of thermal stabilization process. In terms of performance criteria, SEP and CV were used, and clearly suggested that SVR has a better accuracy for the given system identification problem with a small/limited dataset. The developed model was further used for optimization of oxidative stabilization process to reduce the energy consumption, and hence, cost, similar to [26]. The best compromise solution was attained, where the energy savings were up to 48.6% while satisfying the design constraints on the fiber physical (density) property. The study is deemed beneficial for chemical industries involving carbon-fiber manufacturing, with the aim of producing high quality and low cost carbon fiber-based composites with limited dataset for optimization purposes.

The study may be extended by running the oxidative stabilisation tests for different zones of thermal stabilisation oven and investigating the interaction between different zones.

## Figures and Tables

**Table 1:** Neural network-Levenberg-Marquardt algorithm (LMA) configuration model.

Neural-Network Parameters	Levenberg-Marquardt
Number of input parameters	3
Number of hidden neurons	9
Number of output parameters	1
Hidden transfer function	Sigmoidal
Output transfer function	linear
Maximum number of epochs	1000
Learning rate	0.01
Momentum rate	0.9
Stopping gradient	5.5292 × 10^−8^
MU (momentum )	1 × 10^−8^

**Table 2:** The optimum value of parameters in SVR-GA (support vector regression-genetic algorithm) model.

Level	*C*	γ	ε
SVR-GA	3.5739	3.1498	0.0014

**Table 3:** Validation set comparison of the two models.

**Extra Test Point #**		1	2	3	**SEP**	**CV**
**Temperature, °C**		235	233	233		
**Stretching Ration, %**		25	25	30		
**Space Velocity, m/h**		2	4	2		
**ρ_actual_, g·cm^−3^**		1.2580	1.2577	1.2621		
**ANN-LMA**	ρ_predicted_	1.2761	1.2745	1.2756	0.0000149	0.0037
	Error, %	1.4347	1.3336	1.0688		
**SVR-GA**	ρ_predicted_	1.2683	1.2666	1.2654	0.0000117	0.0019
	Error, %	0.8179	0.7073	0.2548		

**Table 4:** Amount of energy consumption for each of the conducted experiments.

Trial #	Temperature, °C	Fiber Space Velocity, m/h	Stretching Ratio, %	Energy Consume, MJ
**1**	238	20	1	6.943
**2**	238	20	1	6.943
**3**	235	20	1	6.925
**4**	241	35	4	6.129
**5**	233	20	1	6.663
**6**	233	20	4	6.663
**7**	239	20	1	7.551
**8**	239	20	1	7.551
**9**	233	20	1	6.663
**10**	237	30	3	4.502
**11**	237	30	3	4.502
**12**	237	35	4	3.859
**13**	239	35	4	4.315

**Table 5:** Optimized operational parameters (*T*, *S*, and σ) and the corresponding minimum energy consumption based on the given constraints, and using the GA optimizer; the SVR predictive modeling was used for density.

Density Constraint, g/cm^3^	*T*, °C	*S*, m/h	σ, %	Predicted Density, g/cm^3^	Actual Density, g/cm^3^	Optimized Energy Consumption, MJ	Maximum Eenergy Consumption, MJ	Density Error, %	Energy Saving, %
1.27 ≤ ρ ≤ 1.29	236.9	24.5	4	1.27	1.29	5.513	10.726	−1.7	−48.6
